# Emerging Trends in the Use of Topical Antifungal-Corticosteroid Combinations

**DOI:** 10.3390/jof8080812

**Published:** 2022-08-01

**Authors:** Dalibor Mijaljica, Fabrizio Spada, Ian P. Harrison

**Affiliations:** Department of Scientific Affairs, Ego Pharmaceuticals Pty Ltd., 21–31 Malcolm Road, Braeside, VIC 3195, Australia; dalibor.mijaljica@egopharm.com (D.M.); fabrizio.spada@egopharm.com (F.S.)

**Keywords:** allylamines, azoles, ergosterol, formulation, fungi, infection, inflammation, itch, miconazole, skin, terbinafine

## Abstract

A broad range of topical antifungal formulations containing miconazole or terbinafine as actives are commonly used as efficacious choices for combating fungal skin infections. Their many benefits, owing to their specific mechanism of action, include their ability to target the site of infection, enhance treatment efficacy and reduce the risk of systemic side effects. Their proven efficacy, and positioning in the treatment of fungal skin infections, is enhanced by high patient compliance, especially when appropriate vehicles such as creams, ointments and gels are used. However, inflammation as a result of fungal infection can often impede treatment, especially when combined with pruritus (itch), an unpleasant sensation that elicits an urge to scratch. The scratching that occurs in response to pruritus frequently accelerates skin damage, ultimately aggravating and spreading the fungal infection. To help overcome this issue, a topical antifungal-corticosteroid combination consisting of miconazole or terbinafine and corticosteroids of varying potencies should be used. Due to their inherent benefits, these topical antifungal-corticosteroid combinations can concomitantly and competently attenuate inflammation, relieve pruritus and treat fungal infection.

## 1. Introduction: The Human Skin and Fungal Infections

Human skin is a dynamic and multifunctional organ that provides a physical barrier against the hostile external environment through its highly organised and intricate landscape [1,2,3]. The skin’s landscape is fundamentally composed of three well-integrated features that encompass the following: (1) a specialised, heterogeneous, interlocking, three-layered interface consisting of the outermost epidermis, middle dermis and innermost hypodermis [1,3,4,5]; (2) indispensable adnexal structures including sweat glands, sebaceous glands and hair follicles [1,3,4,5]; and (3) mechanical elasticity and stability [1,3,5]. Essentially, the skin protects our body from attack by harmful chemicals and pathogens, defends against ultraviolet (UV) radiation and mechanical insults, prevents dehydration and overhydration, controls thermoregulation and allows us to perceive the world around us through a highly innervated surface [3,6]. Although the skin functions defensively to keep harmful microbes out, it simultaneously provides a protective niche for its highly abundant, native resident microbiome [3]—a complex and diverse set of microorganisms, consisting primarily of bacteria [7,8,9] and fungi (mycobiota) [10,11], but also harbours parasites and viruses as well [12]. These skin inhabitants [13] can be commensal, symbiotic, opportunistic, or even pathogenic [3,7,8,9,10,11], and can interact with the skin in numerous ways. Some live predominantly in symbiosis and provide the skin with a variety of benefits (e.g., maintaining skin homeostasis), while others can be deleterious (e.g., lead to opportunistic infection, disease) [8,14], depending on a multitude of intrinsic (e.g., age, genetics, immunity, hydration, sebum levels, metabolism) [9,12] and extrinsic (e.g., hygiene and beauty routine, exposure to chemicals and sunlight, climate) factors [9,11,12,15].

The diverse environments found on the human skin can have a significant effect on the number or type of microorganisms that are found. For example, moist or humid body sites (e.g., the axilla, groin, toe web) usually create favourable conditions for fungal and microbial growth, and are therefore, often colonised by numerous microorganisms, while dry body sites (e.g., the forearms, legs) are not so microbiologically diverse. Similarly, areas of the skin with a considerable number of sebaceous glands (e.g., the head and neck) provide an optimal environment for many lipophilic microorganisms (e.g., the genus *Malassezia*) [12]. In addition, the skin’s microbiome can also be affected by exogenous factors: exposure of the skin to UV radiation from the sun, for instance, can disturb the genetic and structural variability of the skin’s microbiome, resulting in susceptibility to microbial infections or exacerbating already existing symptoms [12].

The numerous fungi that colonise the surface of the skin, including those generally innocuous (e.g., genera *Malassezia*) and those that are pathogenic (e.g., dermatophytes, genera *Candida*), are characterised by phylogenetic divergence [10,11,16,17,18]. Despite their differences, they all have a structurally complex hallmark feature known as the cell wall, which is primarily made of a variety of polysaccharides (e.g., glucans, chitin) and (glyco)proteins (e.g., NO-linked oligosaccharides) [19]. Their cell membrane is structurally quite unique as well, since it contains ergosterol, a major membrane lipid that is absent in animals and plants [20]. The fungal cell wall synthesis and maintenance involve a considerable number of biosynthetic and signaling pathways [19] and it safeguards the contents of the fungal cell from different stresses, gives it rigidity and integrity, and defines its structure and shape [19].

The ‘give-and-take’ interaction between the mycobiota, host skin cells, and the immune system is responsible for maintaining skin health, and a disruption of this delicate balance by altering skin barrier or invasive attack by harmful pathogens can lead to impaired and compromised skin function and subsequently result in fungal infection of the skin, known as mycosis [3]. Overall, there are two types of skin mycoses: (1) the common superficial type, and (2) the less common deep, invasive and systemic type [21,22]. The majority of superficial fungi that reside on the skin, hair and nails degrade keratin—a structural protein responsible for maintaining the skin’s structural stability and integrity [23]—and utilise it as a nutrient source for their growth [24,25,26]. When a fungal cell invades the skin surface—specifically the cornified top layer of the epidermis rich in keratin—, it produces keratinase enzyme, which degrades the tissue and ultimately causes skin inflammation that is often accompanied by pruritus (itch) [24,25,26]. These types of fungi are known as keratinophilic dermatophytes, which include several fungi under the genera *Epidermophyton*, *Microsporum* and *Trichophyton*, and can cause several contagious superficial fungal skin infections (dermatomycoses) of various body regions. These include *Tinea corporis* (ringworm, a localised or solitary ring-like rash on the body), *Tinea pedis* (athlete’s foot, a fungal infection between toes), *Tinea unguium* (fungal infection of the toenails and fingernails—onychomycosis), *Tinea cruris* (jock itch, a fungal infection of the groin area) and *Tinea capitis* (a fungal infection of the head or scalp) [24,25,26,27,28].

Genera *Candida* can cause severe fungal infections such as candidiases. Approximately, twenty species of *Candida* are responsible for human skin infections, and the most common species is the yeast-like fungus, *Candida albicans*, which can cause both superficial and systemic types of fungal infections [25,27,29]. The overgrowth of *Candida albicans* can cause severe mucosal and dermal thrush, nappy rash, and genital infection [25,30]. Hyperkeratosis (thickening of the epidermal outermost layer, the stratum corneum, often associated with a keratin abnormality) and epidermal hypertrophy (increased thickness of the keratinocyte layers) persistent with inflammation are diagnostic symptoms for fungal infection caused by the candida species [25].

Since fungi are classified as eukaryotes and, as such, have a complex cellular structure and organisation; many biochemical and cellular processes take place in a similar way as in human cells [31]. Therefore, it is challenging to develop antifungal/antimycotic drug formulations with high selectivity and efficacy, but low side effects in human cells [22]. However, the two key differences between fungal and human cells are the existence of both a cell wall [19] and ergosterol in fungi [20]. With this in mind, most, if not all, antifungals, including those used in topical vehicle formulations (e.g., creams, ointments, gels) [32,33], are aimed at degrading or disrupting the cell wall or cell membrane (and its individual components) of the fungus to inhibit its key biosynthetic pathways and mechanism of infection, and eventually cause fungal cell death [25]. There are five main classes of antifungals: (1) azoles (e.g., miconazole); (2) allylamines (e.g., terbinafine); (3) polyenes (e.g., nystatin); (4) echinocandins (e.g., caspofungin); and (5) antimetabolites (e.g., flucytosine) that are readily used to combat a spectrum of mycoses including dermatomycoses and candidiases [25,34,35]. Today, these antifungals [22,34,35] are often combined with corticosteroids (see Section 2) of varying potencies [36,37,38] in order to simultaneously alleviate inflammation [39,40,41], diminish the itch-scratch cycle [39,40,41,42], limit spreading and subsequently suppress fungal infection [39,40,41]. The combination of topical antifungal actives with corticosteroids represents a potentially hugely beneficial treatment strategy for fungal infection, as the inflammatory component of the infection can often exacerbate the condition and impede its resolution.

In this review, our primary focus is on the uses, benefits and potential challenges associated with the combination of miconazole or terbinafine with corticosteroids of varying potencies in topical formulations to alleviate inflammation and pruritus, and to treat fungal skin infection. Furthermore, we dissect these miconazole/terbinafine-corticosteroid combinations to their individual actives and indicate how and to what extent these individual active components contribute—be it positively or negatively—to such combinations’ innovation, diversity, formulation and patient compliance, efficacy, and safety.

## 2. Topical Antifungal-Corticosteroid Combinations

### 2.1. Setting the Scene: The Need for Antifungal-Corticosteroid Combinations

**Figure 1:**
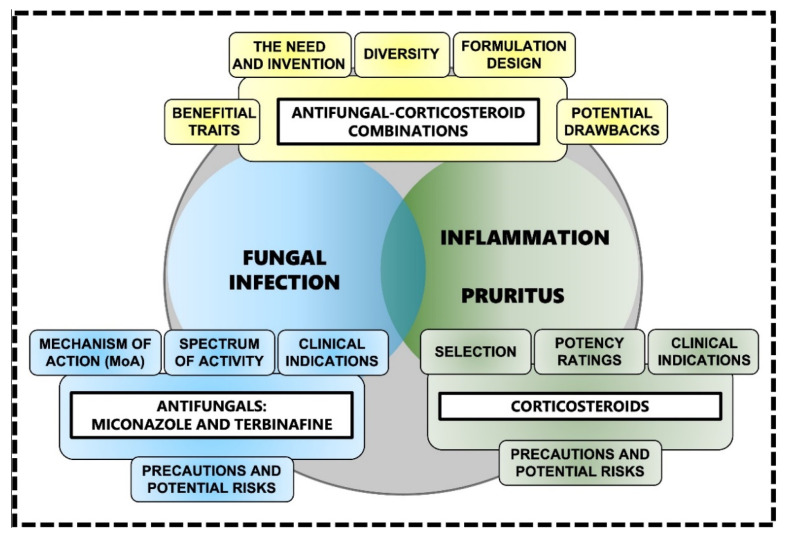
Topical antifungal-corticosteroid combinations as innovative and emerging approaches to the treatment of various fungal infections associated with inflammation and pruritus [39,40,41,45].

During a superficial mycosis or dermatomycosis such as athlete’s foot or jock itch [24,25,26,27,28], the degradation of keratin via the release of an arsenal of hydrolytic enzymes (including keratinases such as subtilisin-like proteases and dipeptidyl peptidases) [43,44] triggers a skin-specific immune response [44]. Subsequently, it results in the release of pro-inflammatory mediators such as tumor necrosis factor (TNF) and interleukin 6 (IL-6) among many others [39,44]. This in turn causes inflammatory symptoms such as pruritus, erythema, swelling and burning at the site of infection. Not only can these symptoms cause substantial discomfort, scratching and even sporadic pain, they can also decrease patient adherence to treatment, ultimately leaving the skin damaged, compromised and susceptible to secondary fungal or bacterial infection, or bacterial superinfection [39]. As such, antifungal treatment strategies should ideally incorporate topical corticosteroids [39,40,41,45] to alleviate the inflammation associated with superficial mycoses [39,40,41,45]. Such strategies [39,40,41,45] should ideally contain an antifungal component to simultaneously treat the fungal infection and a corticosteroid component to manage varying degrees of inflammation and associated pruritus [39,40,41,45] (Figure 1). By reducing inflammation and pruritus, topical corticosteroids can reduce the spread of infection and the risk of secondary infection, and ultimately result in a faster and more desirable clinical outcome [39]. It is plausible that the initial suppression of the immune system caused by topical corticosteroid actives causes the infection to temporarily increase its growth rate, ultimately leading to increased uptake of the antifungal active, which in turn produces a more efficient antifungal action.

### 2.2. The Diversity and Use of Existing Antifungal-Corticosteroid Combinations

Although topical antifungals and corticosteroids are available in a broad range of single-active formulations, there are a relatively small number of topical formulations available that combine the two classes of active, especially for the treatment of superficial dermatomycoses of the feet, scalp, and other regions of the body, for both children and adults [39]. An initial twice-daily application of a topical antifungal plus corticosteroid for 1–2 weeks duration is recommended for localised inflammatory, superficial fungal infections of the body (excluding the areas of the body with thinner skin such as the face and groin) and feet [39]. For mild-to-moderate inflamed infections, an antifungal (preferably azoles such as miconazole and clotrimazole) in combination with a mild-to-moderate corticosteroid such as hydrocortisone or clobetasone is usually sufficient [39,41]. For example, a clotrimazole-hydrocortisone combination is the preferred treatment option in children with inflammatory superficial mycoses due to the tolerability and safety of topical hydrocortisone [39,46]. In fact, the efficacy and safety of hydrocortisone in combination with miconazole has long been established: treatment with Daktacort^®^ cream (containing 2% miconazole plus 1% hydrocortisone) in patients with inflamed skin infections of mycotic or bacterial origin induced a significant improvement in suppressing inflammation and was superior to individual treatments with miconazole or hydrocortisone [47]. For severely inflamed infections, an antifungal (again, preferably azole class as isoconazole) in combination with a potent corticosteroid such as diflucortolone, betamethasone, or clobetasol, is recommended [39,41]. Furthermore, some of these topical combinations also contain antibacterial components such as neomycin and gentamycin to reduce the risk of bacterial superinfection [39,41].

Some studies [39,41,45] have shown that there is no significant difference in the resolution of *Tinea cruris* and *Tinea corporis* infection between treatments using an antifungal-corticosteroid combination (e.g., azole class antifungal-corticosteroid) and an antifungal alone (e.g., azole class) [39,41,45]. However, the key conclusion drawn from all of these studies is that an antifungal-corticosteroid combination demonstrates more rapid therapeutic activity and is more effective in achieving clinical resolution than a topical antifungal alone [39].

### 2.3. Emerging Antifungal-Corticosteroid Combinations: Rationale and Formulation Design 

**Figure 2:**
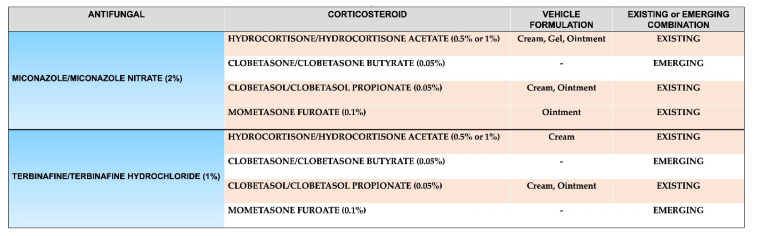
Existing and emerging topical antifungal-corticosteroid formulations containing a combination of azoles (e.g., miconazole) or allylamines (e.g., terbinafine) and selected corticosteroids of varying potency and percentage [39,41,45,51,52].

Due to their superiority to other antifungals, azoles (e.g., miconazole) (see Section 3.1.1 and Section 3.2.1) and allylamines (e.g., terbinafine) (see Section 3.2.1 and Section 3.2.2) are widely used in antifungal-corticosteroid combinations. Both types of antifungals are used at different potencies (e.g., clotrimazole 1%, miconazole/miconazole nitrate 2%, terbinafine/terbinafine hydrochloride 1%) and different dosing regimens [45,48], and both seek to disrupt different parts or intermediate stages of the ergosterol synthesis pathway (see Section 3.1.1 and Section 3.1.2), resulting in changes to the cell membrane that in turn inhibit fungal growth and thus lead to fungal cell death [20,49,50]. The potency of the corticosteroids used in these types of combination can vary (e.g., hydrocortisone/hydrocortisone acetate 0.5% or 1%, clobetasone/clobetasone butyrate 0.05%, clobetasol/clobetasol propionate 0.05%, mometasone furoate 0.1%), so careful consideration should be given to the appropriate potency for the condition, alongside their efficacy and safety profile (see Section 4) [45,51] (Figure 2).

### 2.4. Choosing a Topical Vehicle Formulation for the Delivery of Antifungal-Corticosteroid Combinations

The ideal topical antifungal-corticosteroid combination [39,41,45,51,52] is one that produces a rapid and effective anti-inflammatory response, a high-resolution rate, a low relapse rate, has high patient compliance, a short duration of action, and causes minimal or no adverse effects [45]. The choice of topical antifungal-corticosteroid combination administered to a patient should be specific and tailored according to individual needs and must be used appropriately and according to treatment recommendations and guidelines [39,41]. Single-active vehicle formulations such as creams, ointments and gels that contain either antifungals or corticosteroids alone have been widely used in the treatment of various skin conditions [36,45,48,51,53]. The choice of vehicle formulation will depend on the specific characteristics of the fungal infection (i.e., the body area infected, the total surface area of the infection and degree of inflammation, and the overall severity) on the one hand and the specific characteristics of the patient (i.e., age and the presence of underlying comorbidities, if any) on the other [39]. The most commonly used vehicle formulations for topical antifungal-corticosteroid combinations are creams, ointments and gels [41,45].

#### 2.4.1. Creams

Creams are emulsions of oil dispersed in water, or less commonly water dispersed in oil. They have good hydrating and emollient qualities; their ability to absorb into the superficial layers of the skin and their relatively high water content makes them cosmetically appealing. Creams are generally less efficacious than ointments when it comes to hydrating capacity due to shorter contact time, and also tend to not be the vehicle of choice for topical drug delivery for the same reason. However, creams are useful in intertriginous areas of the body (e.g., the axilla of the arm) where ointments may not be used. However, creams do not provide the occlusive effects that ointments provide [32,33].

#### 2.4.2. Ointments

On the other hand, ointments generally consist of a ‘greasy’ hydrophobic base, usually white soft paraffin, which forms an occlusive layer over the skin. Ointments are effective in enhancing the percutaneous absorption of topical actives by increasing the hydration, and thus barrier function, of the skin. Their long contact time also usually makes them the vehicle of choice for topical drug delivery, including antifungal-corticosteroid combinations. Compared to creams and gels, ointments are generally the least spreadable of the three. The greasy nature of ointments can sometimes limit patient compliance and they are not always cosmetically appealing, particularly on hair-bearing skin [32,33].

#### 2.4.3. Gels

Gels, which include hydrogels, are usually water-based, and consist of transparent lattices of organic macromolecules. They tend to be thick and liquefy on contact with warm skin, providing a pleasant sensation. They dry to form a thin film that does not stain or leave behind a greasy texture and surface evaporation of the water within the formulation creates a cooling effect, which can be beneficial for skin prone to pruritus. These features make gels one of the more cosmetically pleasing topical vehicles. Gels are both easy to apply and wash off. They are particularly suitable for use in sebum-rich oily areas, such as the face, and also in hairy areas of the body [32,33,54].

### 2.5. Misuse of Topical Antifungal-Corticosteroid Combinations

While the combination of topical antifungals with corticosteroids is an obvious choice in terms of treatment efficacy, it does still pose questions around safety, especially if misuse or even overuse occurs due to inadequate training or regulatory guidance on use. In India for example, combination formulations containing topical antifungals and corticosteroids are easily available over the counter (OTC) and are widely used without strict enforcement of existing drug regulations or supervision by a trained healthcare professional [41]. Furthermore, many of these antifungal-corticosteroid combinations are significantly cheaper than single-active antifungal formulations, and provide quick symptomatic relief, meaning they are often the go-to choice, even if the fungal infection being treated does not have any associated inflammation. These combination formulations account for over 50% of the sales of all topical corticosteroids available on the Indian market [41]. Some local or even systemic side effects have been documented, most often linked to misuse such as the prolonged use of medium to high-potency corticosteroids (e.g., clobetasol), especially on the face, or use on large surface body areas [40,41]. However, it seems that the issue is not with these topical combinations themselves and any inherent safety concerns, rather, the issue of safety relates to the education of both patients and healthcare professionals as to appropriate usage, and also regulatory oversight to minimise (or even eradicate if possible) misuse and potential overuse.

By contrast, in Europe where laws and regulations controlling the production and sales of drugs are stringent and are strictly implemented, an expert panel of scientists [39,40] strongly supports the use of antifungal-corticosteroid combinations for the treatment of inflammatory fungal skin infections [39,40,52]. This expert panel has recommended that an antifungal-corticosteroid combination be administered at the beginning of treatment for 1–2 weeks, followed by an appropriate antifungal alone. When used appropriately (which again highlights the importance of education) by immunocompetent patients only (immunocompromised patients should not use such combinations as fungal infection can be more invasive), antifungal-corticosteroids have been shown to be well-tolerated, effective, and more importantly safe in the treatment of superficial inflammatory mycoses [39,40,41].

## 3. Miconazole and Terbinafine: Contributions to Topical Antifungal-Corticosteroid Combinations 

Antifungals (Figure 1) are characterised by a wide variety of chemical structures and a broad range of mechanism of action (MoA) [22,34,35,55,56,57]. As noted earlier, antifungals such as azoles and allylamines interfere with the biosynthesis or integrity of ergosterol [22] while others such as echinocandins cause disruption of the fungal cell wall and its individual components [25,34]. Ergosterol is one of the essential sterols and critical components for the maintenance of fungal cells, as it coordinates membrane heterogeneity, prevents water penetration, and preserves the integrity, rigidity, and fluidity of the cell membrane [22]. Even though ergosterol is somewhat similar to cholesterol that is present in the plasma membrane of animal cells (or sitosterol that is present in plants) [20], antifungals that target ergosterol synthesis or binding do not interact with host cells as there are sufficient and distinct structural differences between ergosterol and cholesterol [20,29].

### 3.1. MoA and Spectrum of Activity

#### 3.1.1. Azoles

The azole antifungal family has a broad spectrum of activity and includes two subclasses on the basis of the number of nitrogen atoms in a five-membered cyclic azole ring. The first class contains two nitrogen atoms in a cyclic azole ring and includes imidazoles (known as first generation azoles) which consist of miconazole, clotrimazole, oxiconazole, econazole, ketoconazole and tioconazole. The second class includes triazoles (known as second generation azoles) such as fluconazole, posaconazole, itraconazole, terconazole, and voriconazole, which contain three nitrogen atoms in a cyclic ring [22,29,58]. Azoles have fungistatic, or at high concentrations fungicidal properties, and can affect fungal cell growth and proliferation by generating a large number of toxic sterols that eventually lead to fungal cell death [34,35,59]. Their potent fungicidal activity and broad spectrum of coverage have made azoles the first-line treatment option for many fungal infections, including dermatomycoses and candidiases [35,60] as well as for systemic mycoses [61].

**Figure 3:**
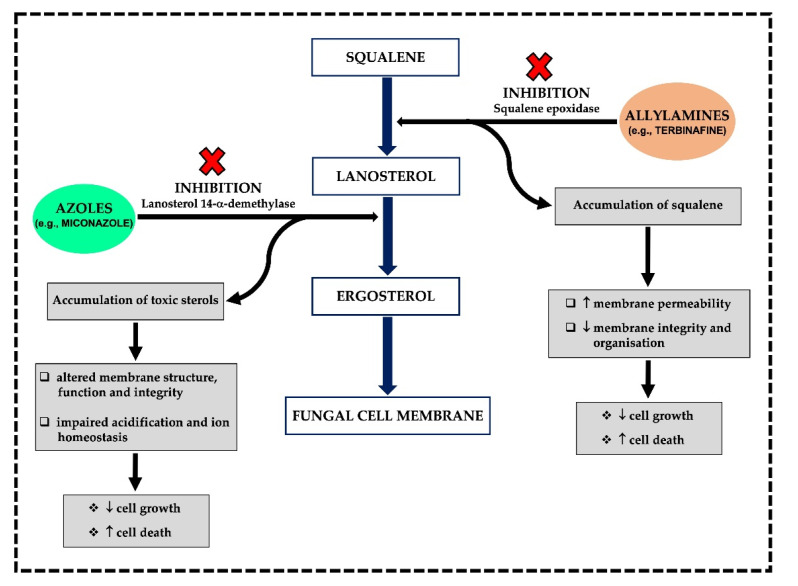
Flow chart showing the MoA of miconazole and terbinafine and their effects on the structure, function and integrity of the fungal cell membrane [20,22,29,34,57,60,62,65,66,67,68].

In the endoplasmic reticulum (ER) of the fungal cell, both azole subclasses work with the same MoA; they interfere with ergosterol biosynthesis (Figure 3). They inhibit the cytochrome P450 dependent enzyme lanosterol 14-α-demethylase (CYP51) by binding to its active site in a non-competitive manner [22,29,62]. CYP51 is present in the outer membrane of the ER and catalyses the removal of the methyl group at carbon 14 [22,29,62]. In simple terms, CPY51 is crucial to the conversion of lanosterol precursor into ergosterol [29,62]. The inhibition of CYP51 by azoles such as miconazole (Figure 3) leads to the accumulation of eburicol and toxic 14-α-methylsterols, including 14-α-methyl-3,6-diol [63,64] which can disrupt the close packing of acyl chains of phospholipids and alter the function of the cell membrane-bound enzymes (e.g., enzymes of the mitochondrial electron transport chain) [57]. As the concentration of ergosterol is reduced and the concentration of lanosterol, eburicol and 14-α-methyl-3,6-diol is increased, the cell membrane structure is altered, which causes the formation of pores in the membrane, making the membrane more permeable and ‘leaky’, and ultimately inhibiting fungal cell growth [29]. Furthermore, depletion of membrane ergosterol by azoles is also known to disrupt vacuolar ATPase functions in the fungal cell membrane, resulting in an impairment of vacuolar acidification and ion homeostasis [34,65].

#### 3.1.2. Allylamines

Allylamines, including terbinafine (Figure 3), naftifine, butenafine and amorolfine [22,66], are a relatively new class of ergosterol biosynthesis inhibitors [60]. This inhibition coincides with accumulation of the sterol precursor squalene and the absence of any other sterol intermediates, suggesting that allylamine inhibition of sterol synthesis occurs at the point of squalene epoxidation (conversion of squalene-to-squalene epoxide) (Figure 3), a reaction catalysed by the enzyme known as squalene epoxidase [22,67,68]. Squalene epoxidase is an essential flavin adenine dinucleotide-dependent enzyme that is not bound to the cytochrome P450 enzyme system [22]. Therefore, fungal cell death is primarily related to squalene accumulation rather than ergosterol deficiency [69]. High levels of squalene can increase membrane permeability, leading to disruption and alteration of the integrity and organisation of the fungal cell membrane, thus influencing its overall structure and function [20,58,67,70].

### 3.2. Miconazole and Terbinafine: Formulation Availability, Clinical Indications and Potential Risks

#### 3.2.1. Miconazole

Miconazole is a synthetic imidazole antifungal that has built a reputation for fast fungicidal action when used topically against a wide range of dermatophytes and *Candida* species [22,71]. Miconazole remains primarily a topical antifungal agent and many patients have been treated with this compound in various formulations. Common indications for topical miconazole include oral and vaginal candidiasis (thrush), dermatomycosis and onychomycosis [25,26,59,71,72,73]. Miconazole antifungals are available most commonly in cream but also in gel, solution, or spray formulations at a 1% [74] or 2% concentration [45] and most are indicated for use twice-daily for 2–4 weeks [25,26,71,72,73] or occasionally for up to 6 weeks if required [75]. More potent miconazole containing topical preparations are also used, such as in the USA, where several products that contain miconazole, typically a 2–4% cream, has been approved for OTC sale [71]. When using miconazole topically, particularly in the form of a cream, serious adverse effects are uncommon; reports of maceration, redness of the skin, and allergic or irritant contact dermatitis are sporadic and rare [22,71,74,76].

#### 3.2.2. Terbinafine

Terbinafine, at low concentrations, has a fungicidal effect on dermatophytes and some other fungi such as moulds. It has a fungistatic or fungicidal effect on yeasts, depending on the species, and whether it is used individually or in combination with other antifungals [22,57]. As terbinafine specifically interferes with fungal sterol biosynthesis in its early stages, therapy is more likely to be of a shorter duration than with other antifungal drugs. When terbinafine is used in cream formulations, the resolution of clinical symptoms usually occurs within a few days, with therapy usually lasting for up to 2 weeks [22], rather than the 2–4 weeks typically found with other azoles. For example, in some early terbinafine clinical trials [77], mycological and overall efficacy rates of around 80% have been achieved in cutaneous dermatophyte infections (*Tinea corporis*, *Tinea cruris* and *Tinea pedis*) with a 1% terbinafine cream applied topically twice-daily [45,77]. Additionally, topical terbinafine has been effective in approximately 80% of patients with cutaneous candidiasis as well [77]. In terms of safety, topical terbinafine has been very well-tolerated in clinical trials to date, with only minor adverse events reported [77] including redness, sensitivity reactions and local irritation [22].

In a 2009 study [78], 1% terbinafine in a topical cream, gel or solution demonstrated significant benefits and efficacy in the management of both *Tinea corporis* and *Tinea cruris*. The application of terbinafine once-daily for 1–2 weeks resulted in mycological cure rates ranging from 84% to 94%, clinical cure rates ranging from 75% to 84% and overall efficacy rates ranging from 65% to 83% [78].

### 3.3. Antifungal Resistance and Alternatives to Conventional Antifungal Treatment Strategies

Fungal cells have developed several resistance mechanisms to counteract the fungistatic and fungicidal effects of antifungals. They have done this primarily through three coexisting and synergistic mechanisms: (1) reduction of antifungal accumulation in the fungal cell, (2) change in the cell’s metabolism that can compromise an antifungal’s efficiency, and (3) increase in antifungal efflux and thus decrease in antifungal affinity [25]. In other words, fungal cell resistance against antifungals is principally associated with the entry of the antifungal active into the cell, its activity on the target cell, its efflux, and lastly its inactivation, degradation, or expulsion from the cell [25,58]. For example, resistance to azoles in dermatomycoses and candidiases [34] and terbinafine resistance in onychomycosis [25] is attributed to the amino sequence modifications of ergosterol biosynthesis proteins that subsequently upregulate their expression. This is turn leads to the development of gain-of-function mutations resulting in a hyper-resistance of fungi to azoles or terbinafine, respectively [25,34].

As a result of increased antifungal resistance, there is a push for alternative, nonconventional antifungal treatment strategies. Examples of these include the use of essential oils extracted from various plants or plant parts (e.g., thyme, peppermint, clove), and device-based treatments such as photodynamic therapy [28,79]. Essential oils are rich in terpenes and phenolic compounds that can exert antimicrobial and antifungal properties, including loss of fungal membrane integrity, reduction of ergosterol levels and inhibition of cell wall formation. Even though it is widely anticipated that essential oils in optimised topical vehicles such as creams, ointments and gels may eventually replace conventional antifungal formulations, the mechanisms of action of essential oils is not well understood [80]. Photodynamic therapy uses a narrow-spectrum light source to activate topically applied photosensitisers to damage and kill fungal cells [79]. While devices such as these are considered alternatives to conventional antifungal treatments in places such as the USA, they are only approved for cosmetic applications, not to cure infection [79]. With time and research, both strategies may prove to be efficacious alternatives to traditional antifungals, but until then, the best antifungal treatment strategy remains the use of topical antifungal actives, in combination with corticosteroids (see Section 4).

## 4. The Contributions of Corticosteroids to Topical Antifungal-Corticosteroid Combinations

There is an abundance of topical corticosteroids available in a variety of potencies and formulations [36,37,38,53,81,82,83]. It is important to indicate that desirable and successful corticosteroid treatment depends on a range of parameters: (a) accurate diagnosis; (b) consideration of the corticosteroid’s delivery vehicle formulation; (c) potency; (d) frequency of application; (e) duration of treatment; (f) effectiveness; and (g) safety and potential adverse effects [36] (Figure 1).

### 4.1. Potency Ratings/Classification

Based on their skin vasoconstrictive activity, topical corticosteroids are classified in two different ways by the British National Formulary (BNF) and the USA classification systems [81], as per the Stoughton-Cornell classification framework [84]. The USA classification system includes seven potency classes (numbered Classes 1–7 in descending order of potency; Class 1: superpotent/very potent to Class 7: least potent/mild), while the British classification system contains four classes (numbered Classes I–IV again in descending order of potency; Class I: superpotent/very potent to Class IV: least potent/mild [51,53,85,86]. According to the USA classification system, it is important to note that the greater the potency of topical corticosteroids, the greater the expected therapeutic efficacy and thus side effects [81]. As such, best-practice dictates that low-potency formulations should be used for longer-term (usually up to 4 continuous weeks) treatments while more potent options should be chosen for short periods of time and body sites with a higher skin thickness such as the palms and bottoms of the feet, where low potency topical corticosteroids take longer to absorb and are less effective [81,87,88]. Under the USA classification system, the potency of a product is defined by the topical corticosteroid used and its concentration as well as the topical vehicle formulation used. On the other hand, the BNF classification system does not take into account the vehicle formulation that is used [81].

Australasian guidelines favour the BNF classification system [51,53,86] of four classes; (however, in ascending order of potency) Class I: mild to Class IV: very potent [82,86]. According to the Australasian guidelines, the choice of potency will largely be guided by the following factors: (1) patient’s age—children often require a shorter duration of treatment and a lower potency corticosteroid; (2) body site of application, and skin condition and severity; (3) topical corticosteroid used (e.g., molecular structure, percentage and formulation) and amount (e.g., by using the fingertip unit method); and (4) method of application (e.g., occlusive dressing versus wet dressing; occlusive dressing increases potency with better efficacy and effect, whereas wet dressing intensifies the effect by improved permeability of topical corticosteroid) [38]. Such guidelines should be strictly followed as indicated for a particular product in order to minimise and even completely avoid any adverse effects [89].

### 4.2. Selecting Formulations, Clinical Indications and Potential Risks

The specific molecular structure of a corticosteroid, the formulation used, and the concentration of active, all affect the potency of a corticosteroid. In addition, other variables such as the body application site or use of a dressing may alter the effect of the corticosteroid. Generally speaking, the actions of a corticosteroid are impacted in accordance with the following principles: (a) ointments penetrate skin slightly better than creams as they occlude the skin and enhance hydration and absorption [38]; (b) thin skin (e.g., the face and eyelid) absorb corticosteroids more readily than areas with thick skin (e.g., the trunk, hand and foot) [38,53]; and (c) intertriginous areas (e.g., the axilla, groin and breast folds) retain topical corticosteroids longer, with increased absorption [38].

Adverse effects are rare if topical corticosteroids are used correctly and according to established recommendations and guidelines. However, misuse or prolonged use of topical corticosteroids may cause some adverse effects [36,38,90,91]. To reduce the risk, the least potent corticosteroid should be used for the shortest time, while still maintaining effectiveness. The most common side effect of topical corticosteroid use is skin atrophy. All topical corticosteroids can induce skin atrophy, but the use of higher potency corticosteroids, occlusion of the skin, thinner skin, and age can all increase the risk. Other common adverse effects from topical corticosteroid use include striae (stretch marks) and telangiectasia characterised by small, widened blood vessels [36,38,90,91].

## 5. Conclusions

Topical antifungal formulations containing a single active such as miconazole or terbinafine can be usually effective in the treatment of dermatomycoses and candidiases. However, if inflammation and itching are present, initial short-term treatment with an antifungal-corticosteroid dual-active combination may be beneficial, followed by treatment with a topical antifungal alone if required. This will result in a faster onset of antifungal action and a faster resolution of inflammatory symptoms and associated itch. Such swift symptom relief may also lead to increased adherence to treatment—easing of inflammation and itch by corticosteroids might be interpreted as a ‘cure’—and may reduce the risk of an aggravated and prolonged fungal infection, potentially accompanied by bacterial superinfection. A diverse range of existing and emerging antifungal-corticosteroid combinations can be considered, as they should be indispensable in situations where inflammatory symptoms are observed most frequently, such as in infections caused by diverse dermatophytes. The choice of antifungal-corticosteroids combination and treatment regimen will depend on the specific characteristics of the infection and the patient.

## Data Availability

Not applicable.
